# Succinate metabolism: a promising therapeutic target for inflammation, ischemia/reperfusion injury and cancer

**DOI:** 10.3389/fcell.2023.1266973

**Published:** 2023-09-22

**Authors:** Wenhui Zhang, Ren Lang

**Affiliations:** Department of Hepatobiliary Surgery, Beijing Chao-Yang Hospital Affiliated to Capital Medical University, Beijing, China

**Keywords:** succinate, succinate dehydrogenase (SDH), immune cells, inflammation, ischemia/reperfusion (IR) injury, cancer

## Abstract

Succinate serves as an essential circulating metabolite within the tricarboxylic acid (TCA) cycle and functions as a substrate for succinate dehydrogenase (SDH), thereby contributing to energy production in fundamental mitochondrial metabolic pathways. Aberrant changes in succinate concentrations have been associated with pathological states, including chronic inflammation, ischemia/reperfusion (IR) injury, and cancer, resulting from the exaggerated response of specific immune cells, thereby rendering it a central area of investigation. Recent studies have elucidated the pivotal involvement of succinate and SDH in immunity beyond metabolic processes, particularly in the context of cancer. Current scientific endeavors are concentrated on comprehending the functional repercussions of metabolic modifications, specifically pertaining to succinate and SDH, in immune cells operating within a hypoxic milieu. The efficacy of targeting succinate and SDH alterations to manipulate immune cell functions in hypoxia-related diseases have been demonstrated. Consequently, a comprehensive understanding of succinate’s role in metabolism and the regulation of SDH is crucial for effectively targeting succinate and SDH as therapeutic interventions to influence the progression of specific diseases. This review provides a succinct overview of the latest advancements in comprehending the emerging functions of succinate and SDH in metabolic processes. Furthermore, it explores the involvement of succinate, an intermediary of the TCA cycle, in chronic inflammation, IR injury, and cancer, with particular emphasis on the mechanisms underlying succinate accumulation. This review critically assesses the potential of modulating succinate accumulation and metabolism within the hypoxic milieu as a means to combat various diseases. It explores potential targets for therapeutic interventions by focusing on succinate metabolism and the regulation of SDH in hypoxia-related disorders.

## 1 Introduction

Succinate and succinate dehydrogenase (SDH) are essential components of the tricarboxylic acid (TCA) cycle and are integral to the production of adenosine triphosphate (ATP) within mitochondria (Murphy and O'Neill, 2018). Additionally, succinate serves as a critical participant in various metabolic pathways, contributing to the regulation of numerous catabolic and anabolic processes within different stages of the cycle (Mills and O'Neill, 2014).

Over the past decade, there has been a growing recognition of the capacity of immune cells to adapt their metabolic processes in order to maintain immune homeostasis in hypoxic environments. These metabolic alterations have been found to have significant implications for the immune response to chronic inflammation, ischemia/reperfusion (IR) injury, and tumors (Fulcher and Katelaris, 1991; Murphy and Chouchani, 2022). Recent literature has increasingly highlighted the non-canonical functions of succinate levels and the regulation of SDH beyond their traditional role in metabolism. For instance, succinate has been found to stimulate dendritic cells (DCs) and stabilize hypoxia-inducible factor-1α (HIF-1α) via the succinate receptor, G protein-coupled receptor 91 (GPR91), in distinct tumors and activated macrophages (Mills and O'Neill, 2014; O'Neill and Pearce, 2016; Huang and Millar, 2013; Dalla Pozza et al., 2020). Furthermore, mutations in the SDH gene have been detected in various cancer types, suggesting potential mechanisms that lead to abnormal succinate accumulation (Peti-Peterdi, 2010). Succinate can function as an oncometabolite in tumorigenesis and cancer progression, and it can also induce post-translational modifications (PTMs) of proteins through succinylation (Mills and O'Neill, 2014; Mills et al., 2021). This review offers a comprehensive analysis of the roles played by succinate and the changes in SDH expression or activity that are linked to modifications in immune cell function during hypoxic conditions. Alongside summarizing the pathways leading to succinate accumulation, our objective is to elucidate potential therapeutic targets by focusing on succinate metabolism in the context of inflammation, IR injury, and cancer.

## 2 Succinate and SDH in metabolism

### 2.1 Biogenesis of succinate

Succinate, or butanedioic acid, was initially isolated and identified from amber through the process of dry distillation by Georgius Agricola, a German chemist, in 1,546 (Tretter et al., 2016). One of the most significant scientific breakthroughs of the 20th century was the elucidation of the TCA cycle, also known as the citric acid cycle, by Hans Adolf Krebs. In 1937, Krebs established that the succinate-fumarate-malate-oxaloacetate pathway constituted a vital component of the TCA cycle, with the inclusion of citrate, isocitrate, and α-ketoglutarate (α-KG) (Raju, 1999). For decades, the TCA cycle has been extensively employed in the microbial synthesis of citrates, glutamates, and succinates (Vuoristo et al., 2016). In the TCA cycle, succinate is synthesized through the catalytic action of succinyl-CoA synthetase from succinyl-CoA. Subsequently, succinate is promptly converted to fumarate by the enzyme SDH. In summary, succinate and SDH are of paramount importance in ATP generation and serve as pivotal cyclic pathways in metabolism, functioning as a reservoir for catabolic processes and as a point of origin for various anabolic processes (Chinopoulos, 2013).

### 2.2 Roles of SDH in the TCA cycle and ETC

Succinate and SDH exhibit a robust interconnection within the TCA cycle and electron transport chain (ETC.). SDH, referred to as complex II, facilitates the integration of two significant pathways within mitochondria, namely, the oxidation of succinate to fumarate as a vital constituent of the TCA cycle and the transformation of ubiquinone to ubiquinol in the mitochondrial, ETC. Both processes are indispensable for oxidative phosphorylation, which is responsible for ATP generation to sustain biogenesis (Gill, 2012; Tretter et al., 2016).

The structure of SDH is characterized by its intricate composition of multiple subunits, namely, SDHA, SDHB, SDHC, and SDHD. In addition, SDHAF1 and SDHAF2 function as assembly factors for the associated assembly process (Moosavi et al., 2019). Notably, SDHC and SDHD possess hydrophobic properties that enable their anchoring in the inner mitochondrial membrane (Her and Maher, 2015). Conversely, SDHA and SDHB extend into the matrix. SDHA is covalently linked to a flavin adenine dinucleotide (FAD) prosthetic group and harbors the binding site for succinate. In the presence of succinate, SDHA facilitates the oxidation of succinate to fumarate by bringing succinate into close proximity with the isoalloxazine ring of FAD (Hagerhall, 1997). SDHB serves as a connecting link between SDHA and SDHC and SDHD. Within SDHB, three Fe-S centers are present, which facilitate the transfer of electrons from succinate to ubiquinone for utilization in the aerobic and energy-generating respiratory chain in eukaryotic mitochondria and various prokaryotes (Yankovskaya et al., 2003). The Fe-S centers, as subunit components of SDH, offer new perspectives for the development of candidate vaccines aimed at inducing anti-embryonation and anti-fecundity immunity (Yu et al., 2007). The SDH complex is comprised of two ubiquinone-binding sites (Yankovskaya et al., 2003; Sun et al., 2005). Residues originating from SDHB, SDHC, and SDHD, which are situated in close proximity to the matrix side of the inner mitochondrial membrane, contribute to the formation of the high-affinity binding sites, thereby enhancing their efficiency (Silkin et al., 2007; Szeto et al., 2007). Conversely, the low-affinity binding site, located nearer to the intermembrane space of the inner mitochondrial membrane, is formed by SDHC and SDHD (Her and Maher, 2015). Consequently, the oxidation of succinate to fumarate by SDH results in the generation of flavin adenine dinucleotide, reduced (FADH_2_). In the, ETC., only SDH (complex II) is completely encoded in nuclear deoxyribonucleic acid (DNA), and electrons from succinate oxidation are transferred to ubiquinone in the, ETC (Vuoristo et al., 2016).

Moreover, ATP can also be generated through substrate-level phosphorylation (SLP). Succinyl-CoA and adenosine diphosphate (ADP) [or guanosine diphosphate (GDP)] undergo catalysis by succinyl-CoA synthetase, resulting in the production of succinate, CoASH, and ATP [or guanosine triphosphate (GTP)]. This metabolic process, referred to as SLP, is predominantly utilized in mitochondria to generate ATP (Johnson et al., 1998). Notably, SLP in the TCA cycle, facilitated by succinyl-CoA ligase (SUCL), operates independently of the respiratory chain and the mitochondrial proton motive force. This ATP production mechanism is in addition to oxidative phosphorylation (OXPHOS) and adenylate kinase (AK) reactions (Bruns and Regina, 1977; Johnson et al., 1998; Nobumoto et al., 1998; Panayiotou et al., 2014; Komlodi and Tretter, 2017). In hence, comprehending the significance of SDH and succinate in metabolic processes can offer novel perspectives for the formulation of therapeutic approaches aimed at addressing inflammation, IR injury, and cancer (Hagerhall, 1997).

## 3 Succinate as a metabolic signal in immuno-inflammatory response

Significant research findings intriguingly indicate that succinate may serve as a novel category of regulators in inflammation, acting as crucial signals to modulate the inflammatory process (Mills and O'Neill, 2014; Tannahill et al., 2013; Grimolizzi and Arranz, 2018; Palsson-McDermott and O'Neill, 2013). Succinate is increasingly recognized as a significant signal in the immuno-inflammatory response.

### 3.1 Stabilization of HIF-1α in inflammation

**FIGURE 1:**
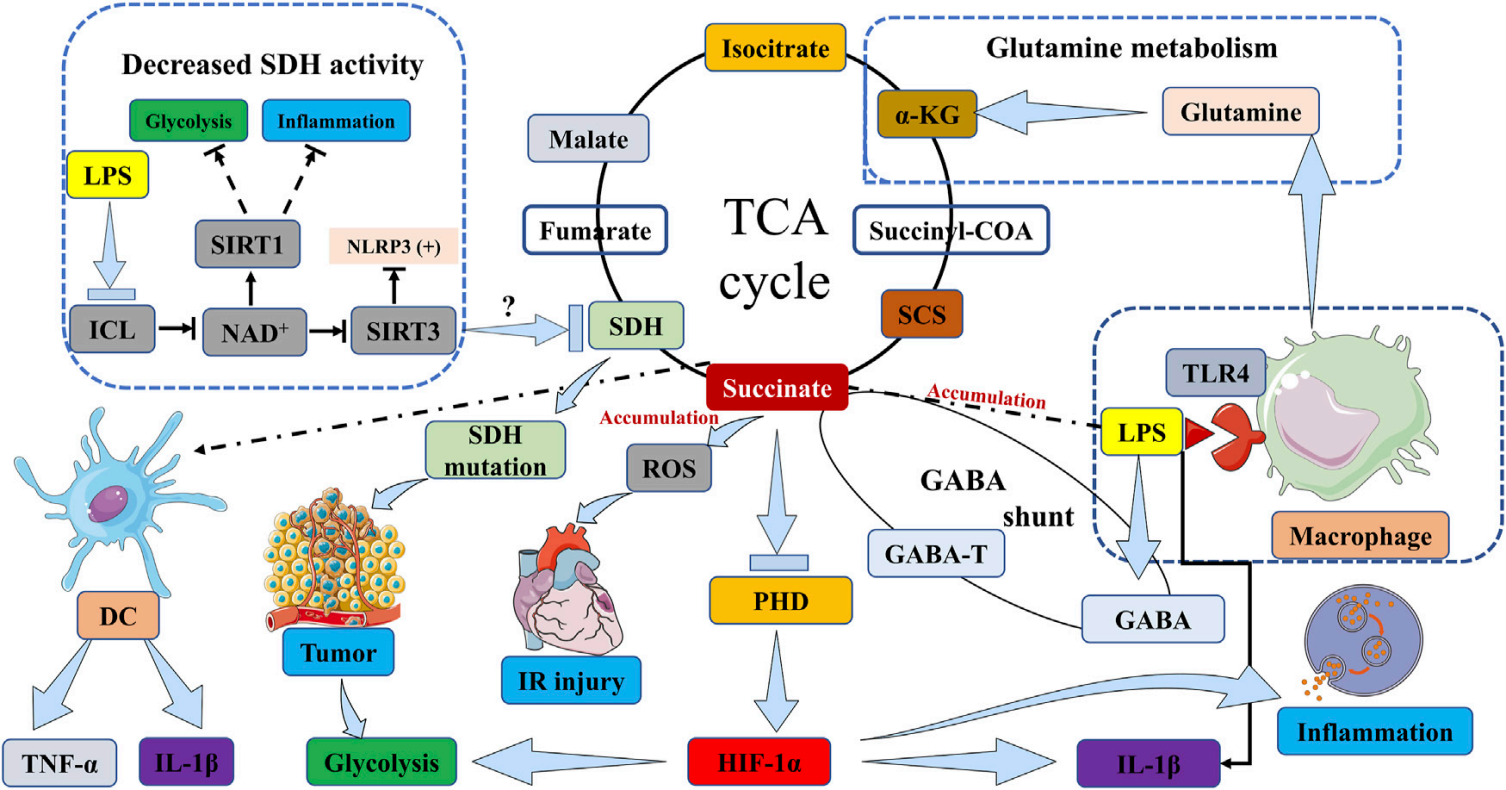
Succinate accumulation pathways in immune cells, inflammation, IR injury and tumor. The activation of macrophages by LPS induces TLR4 signaling, which subsequently initiates the accumulation of succinate. This accumulation of succinate in activated macrophages stabilizes HIF-1α, particularly in the presence of inhibited PHD enzyme activity, thereby resulting in the production of IL-1β through HIF-1α signaling. In DCs, the stimulation of succinate and LPS leads to an increase in the expression of IL-1β and TNF-α. In the context of tumorigenesis, mutations in the SDH gene lead to the accumulation of succinate, which in turn stabilizes HIF-1α, thereby promoting tumor growth and exacerbating inflammation through the upregulation of glycolytic enzymes and IL-1β. Simultaneously, succinate accumulation inhibits tumor cell growth and survival. This accumulation of succinate can occur through two pathways: glutamine metabolism, which promotes the replenishment of α-KG in the TCA cycle, or the “GABA shunt” pathway, which is activated by elevated levels of GABA and its transporters induced by LPS. The inhibition of GABA-T can effectively prevent the accumulation of succinate. The transformation of isocitrate into succinate within the glyoxylate shunt is facilitated by ICL, and this process can lead to an increase in succinate levels. Furthermore, the accumulation of succinate can be attributed to the suppression of SDH activity, which can be triggered by various factors including hypoxia or reduced levels of NAD^+^ induced by LPS. SIRT1 suppresses glycolysis and inflammation. Conversely, the inactivation of SIRT3 has been observed to heighten the activation of NLRP3 inflammasome. ICL, isocitrate lyase; NAD^+^, nicotinamide adenine dinucleotide; SIRT, Sirtuin; LPS, lipopolysaccharide; SDH, succinate dehydrogenase; TCA cycle, tricarboxylic acid cycle; α-KG, α-ketoglutarate; DC, dendritic cell; TNF-α, tumor necrosis factor α; IL-1β, interleukin 1β; PHD, prolyl hydroxylase domain; HIF-1α, hypoxia-inducible factor-1α; SCS, succinyl-CoA synthetase; GABA, γ-aminobutyric acid; GABA-T, γ-aminobutyric acid transaminase; TLR4, toll-like receptor 4. IR injury, ischemia/reperfusion injury; NLRP3, NOD-like receptor family, pyrin domain containing 3.

In hypoxic inflammatory microenvironments, the activation of immune cells, including macrophages and DCs, induces a metabolic shift towards glycolysis (Mills and O'Neill, 2014; Tannahill and O'Neill, 2011). This metabolic shift towards glycolysis in activated immune cells is thought to have a significant impact in low oxygen conditions, such as hypoxic inflammatory sites. The accumulation of succinate has the potential to enhance inflammatory signaling and greatly influence the immuno-inflammatory response. Various potential sources may contribute to the accumulation of succinate. Firstly, these studies have provided confirmation that succinate accumulation resulting from SDH mutations has the ability to stabilize HIF-1α in activated macrophages, particularly when the activity of the prolyl hydroxylase domain (PHD) enzyme is inhibited. This stabilization subsequently leads to the production of the proinflammatory cytokine interleukin 1β (IL-1β) through the signaling of HIF-1α (Mills and O'Neill, 2014; O'Neill and Pearce, 2016; Tannahill et al., 2013; Zhang et al., 2021; Peace and O'Neill, 2022; Williams and O'Neill, 2018) (Figure 1). In addition, it has been observed that succinate accumulation can also occur in lipopolysaccharide (LPS)-activated macrophages through the process of glutamine metabolism, which facilitates the anaplerosis of α-KG into the TCA cycle (Rubic et al., 2008) (Figure 1). Subsequently, the “GABA shunt” pathway can lead to the accumulation of succinate, as it is stimulated by the increased levels of γ-aminobutyric acid (GABA) and its transporters induced by LPS (Figure 1). This pathway can be hindered by vigabatrin which inhibits an essential enzyme of the GABA shunt, resulting in a reduction in succinate accumulation (Figure 1). Additionally, the inhibition of GABA transaminase (GABA-T), a critical enzyme of the GABA shunt, with vigabatrin can effectively prevent succinate accumulation (Tannahill et al., 2013; Ren et al., 2019) (Figure 1). Finally, succinate accumulation can arise from the inhibition of SDH activity during hypoxia and various other pathways. In addition to hypoxia, FAD-induced decrease in nicotinamide adenine dinucleotide (NAD^+^) levels caused by LPS also hampers SDH activity (Wang et al., 2021) (Figure 1). NAD^+^ demonstrates multiple anti-inflammatory properties through the activation of sirtuins, a group of NAD^+^-dependent deacetylases. Specifically, sirtuin 1 (SIRT1) suppresses glycolysis and the activity of proinflammatory transcription factors such as nuclear factor-κB (NF-κB) and HIF-1α (Schug et al., 2010; Misawa et al., 2013), while also promoting autophagy-related gene function and oxidative metabolism. Conversely, the inactivation of SIRT3 has been observed to heighten the activation of the NOD-like receptor family, pyrin domain containing 3 (NLRP3) inflammasome (Schug et al., 2010; Misawa et al., 2013) (Figure 1).

Succinate additionally governs the immune functions of immune cells, specifically pertaining to immune cell migration, cytokine production, and augmentation of the ability of antigen-presenting cells (APCs) to elicit adaptive immune responses (Peace and O'Neill, 2022). For instance, succinate has been found to induce migration of monocyte-derived dendritic cells (MoDCs) *in vitro* and act as a chemokine, thereby promoting migration to draining lymph nodes in succinate-treated DCs (Rubic et al., 2008; Haffke et al., 2019). Succinate has been found to facilitate the production of cytokines through synergistic interactions with certain ligands, including tumor necrosis factor-α (TNF-α) (Rubic et al., 2008). Furthermore, succinate stimulation with LPS has been shown to increase the expression of IL-1β in murine bone marrow-derived dendritic cells (BMDCs), in addition to TNF-α (Williams and O'Neill, 2018) (Figure 1). In addition to promoting cytokine generation, succinate can also enhance the ability of APCs to induce immune responses that result in inflammatory outcomes. Multiple studies have provided evidence of succinate’s capacity to hinder tumor growth, which will be further expounded upon in the subsequent section (Dalla Pozza et al., 2020). Of greater significance, the manipulation of succinate metabolism holds potential as a microbiological therapy in host-microbe interactions (Wei et al., 2023). These findings suggest that the manipulation of succinate metabolism could be employed to devise innovative therapeutics for the prevention and treatment of inflammation.

### 3.2 Succinate and SUCNR1 signaling during inflammation

**FIGURE 2:**
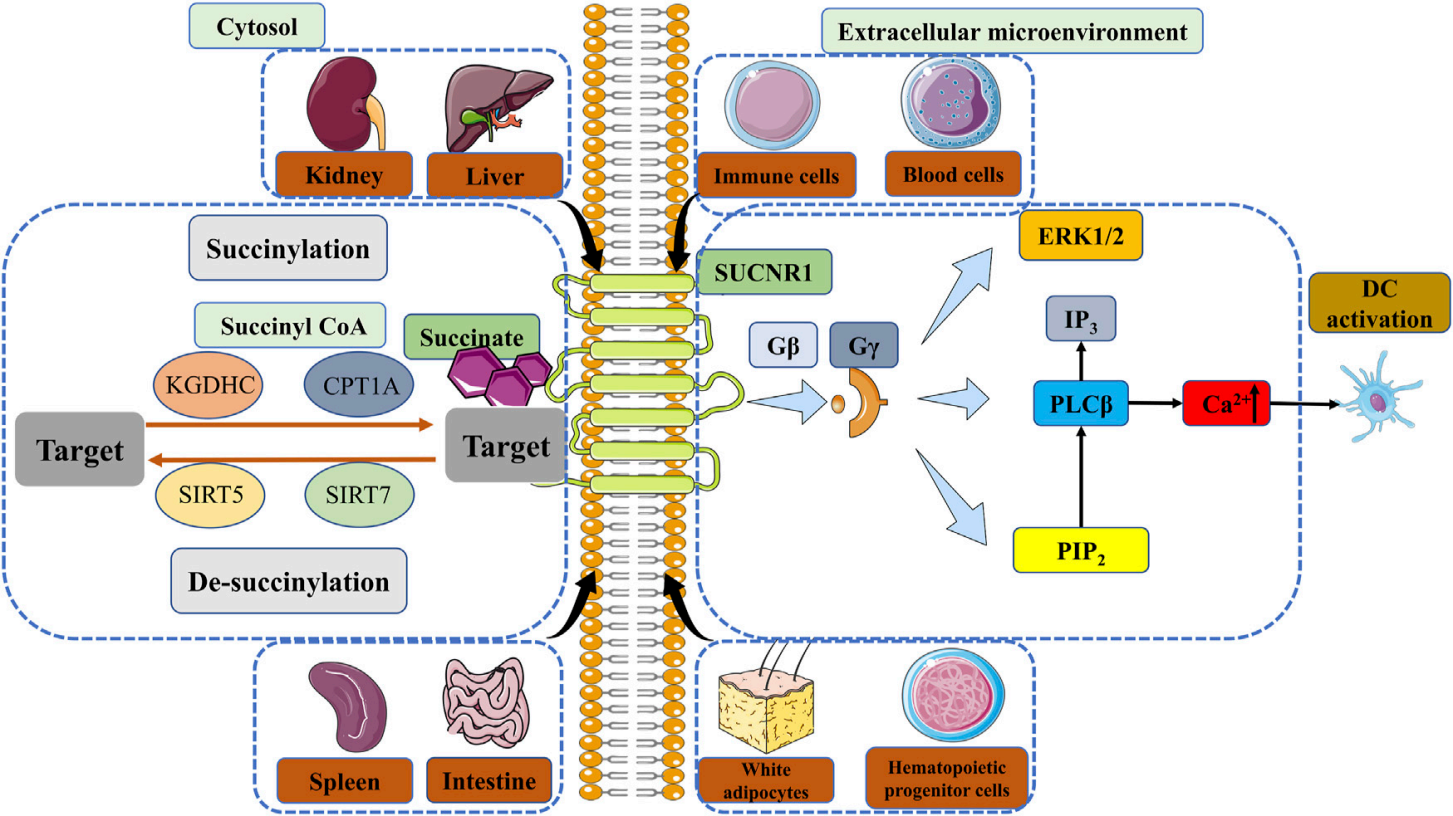
The regulation of succinylation, desuccinylation and the signal of SUCNR1. Enzymatic succinylation is facilitated by succinyl CoA and can be enhanced by KGDHC in the cytosol. CPT1A has the ability to augment lysine succinylation without altering succinyl CoA levels. The levels of SIRT5 have an impact on desuccinylase activity, resulting in modifications in succinylation. Desuccinylation catalyzed by SIRT7 primarily occurs in the nucleus and plays a crucial role in the response to DNA damage and cell survival. Succinate in the extracellular microenvironment can transmit signals through GPR91, thereby sustaining cytokine production and activating DCs. The comprehension of enzymatic succinylation and desuccinylation pathways and mechanisms elucidates the potential of succinate metabolism as a therapeutic approach for the treatment of diverse diseases, such as inflammation, IR injury and cancer. KGDHC, α-ketoglutarate dehydrogenase complex; CPT1A, carnitine palmitoyltransferase 1A; SIRT, Sirtuin; SUCNR1, succinate receptor 1; GPR91, G protein-coupled receptor 91; ERK, extracellular regulated kinase; IP_3_, inositol trisphosphate; PIP_2_, phosphatidylinositol (Fulcher and Katelaris, 1991; O'Neill and Pearce, 2016) bisphosphate; PLCβ, phospholipase Cβ; Ca^2+^, calcium ion; DC, dendritic cell.

There have been numerous studies demonstrating the interaction between the ligand-receptor pair succinate receptor 1 (SUCNR1) (formerly known as GPR91) and succinate, beyond its role as a metabolic signal in inflammation by stabilizing HIF-1α (de Castro Fonseca et al., 2016; Ristic et al., 2017). Wittenberger et al. initially discovered several G-protein coupled receptors, including SUCNR1 (Wittenberger et al., 2001). He et al. later identified succinate as a selective ligand of SUCNR1 (He et al., 2004). SUCNR1 was initially identified and studied in the renal system, subsequently revealing significant expression levels in the hepatic, splenic, and intestinal tissues (He et al., 2004; de Castro Fonseca et al., 2016; Gilissen et al., 2016). SUCNR1 expression has been observed in white adipocytes, hematopoietic progenitor cells (Rubic et al., 2008), as well as various blood and immune cell types (Macaulay et al., 2007; Hakak et al., 2009; de Castro Fonseca et al., 2016; Krzak et al., 2021). Succinate induces the activation of SUCNR1, which subsequently initiates signaling cascades via multiple pathways. Comparable to Gβ and Gγ signaling cascades, succinate stimulation results in the mobilization of calcium ions (Ca^2+^), accumulation of inositol trisphosphate (IP_3_), and phosphorylation involving extracellular regulated kinase (ERK) (He et al., 2004; Robben et al., 2009; Gilissen et al., 2016) (Figure 2). Gaining a comprehensive understanding of the signaling pathways triggered by the interaction between SUCNR1 and succinate can offer novel perspectives for the advancement of therapeutic approaches targeting specific diseases, particularly those associated with chronic inflammation.

Recent studies have provided evidence indicating the involvement of SUCNR1 in multiple succinate-dependent inflammatory processes *in vivo*. Firstly, it was observed that SUCNR1-deficient mice exhibited heightened migration of DCs in comparison to their wild-type counterparts. Secondly, the absence of SUCNR1 in DCs resulted in the absence of cytokine elevation, thereby supporting the notion that succinate functions as a conventional signal to enhance the antigen-presenting function of APCs. Lastly, solid organ transplantation from SUCNR1-deficient mice exhibited prolonged graft survival when compared to that from wild-type mice (Rubic et al., 2008). Peruzzotti-Jametti et al. have presented significant evidence demonstrating that the inhibition of SUCNR1 leads to a reduction in carrier-mediated succinate uptake. This suggests that the signaling of SUCNR1 promotes the expression of plasma membrane Na^+^-dependent dicarboxylic acid transporters, which facilitate the transportation of succinate across the membrane. These findings introduce intriguing possibilities regarding the involvement of succinate in the paracrine/autocrine regulation of disease development (Peruzzotti-Jametti et al., 2018). To sum up, the aforementioned findings have indicated that SUCNR1 plays a pivotal role in the immune-inflammatory response triggered by succinate. Consequently, the modulation of SUCNR1 signaling presents a promising therapeutic strategy for the management of inflammatory disorders.

### 3.3 Succinate and succinylation in signal transduction

Succinylation, a PTM mechanism, entails the incorporation of a succinyl group onto a protein residue, predominantly lysine residues, thereby influencing the functionality of amino groups (Hirschey and Zhao, 2015; Alleyn et al., 2018; Sreedhar et al., 2020; Chinopoulos, 2021). Recent investigations have concentrated on the succinylation modification, wherein succinyl CoA can form an amide bond with protein lysine (Zhao et al., 2023). The identification of elevated succinate levels in numerous proteins involved in the regulation of diverse cellular and biological processes underscores the significance of succinylation as a crucial biological function (Xie et al., 2012; Park et al., 2013; Hirschey and Zhao, 2015). The catalytic processes involved in succinylation mediated by multiple enzymes, remain poorly understood. Consequently, there is ongoing research aimed at elucidating these fundamental mechanisms in order to facilitate the development of innovative therapeutic interventions and associated pharmaceuticals. Numerous investigations have demonstrated that succinylation can occur in organisms through both enzymatic and non-enzymatic means. In fact, the majority of is known to occur through non-enzymatic processes, as extensively documented in the literature (Sreedhar et al., 2020; Shen et al., 2023; Zhao et al., 2023), particularly by Matthew D Hirschey et al., who have demonstrated the higher chemical reactivity of succinyl-CoA compared to other acyl-CoA species (Wagner et al., 2017). Non-enzymatic succinylation appears to be influenced by various factors, including the concentration-dependent levels of reactants within mitochondria, while the catalysis of enzymatic succinylation may be regulated by succinyl CoA. Enzymatic succinylation may be promoted via α-ketoglutarate dehydrogenase complex (KGDHC) and E1k [α-Ketoglutarate Dehydrogenase (KGDH)] beyond succinyl CoA (Yang and Gibson, 2019) (Figure 2). Succinylation is consistently mediated by KGDHC in the cytosol and nucleus. Enzymatic TCA cycle steps like succinyl-CoA synthetase (SCS) can also regulate succinyl-CoA levels, which then non-enyzmatically drive succinylation (Li et al., 2015; Carrico et al., 2018; Gut et al., 2020). The carnitine palmitoyltransferase 1A (CPT1A) G710E mutant has been observed to enhance cell proliferation in the presence of metabolic stress, while CPT1A increases lysine succinylation without any changes in succinyl CoA levels in mammalian cells (Kurmi et al., 2018; Wang et al., 2019). This finding suggests that the lysine succinyltransferase activity of CPT1A may succinylate downstream substrate proteins, thereby facilitating proliferation (Kurmi et al., 2018; Sreedhar et al., 2020). In contrast to the established mechanism of succinyl-CoA non-enzymatic lysine succinylation, the mechanism promoted by CPT1A remains controversial and has not been shown *in vivo* (Sreedhar et al., 2020; Shen et al., 2023). At best, this mechanism would account for a minor fraction of succinylation (Sreedhar et al., 2020). The potential impact of concentration-dependent acyl-CoA distribution on succinylation in certain tissues has been identified (Weinert et al., 2013). This finding implies that the tissue-specific regulation of acyl-CoA concentrations offers novel therapeutic strategies for modulating succinylation in certain diseases. Furthermore, the regulation of de-succinylation plays a crucial role in controlling various catalytic processes across all cellular compartments, with succinylation potentially exhibiting a balanced reciprocal relationship. Notably, the levels of NAD^+^-dependent SIRT5 have been found to influence dynamic changes in desuccinylase activity (Figure 2). Certain studies have provided evidence indicating that the inhibition of SIRT5 results in an elevation of succinylation in particular proteins, thereby contributing to the development and progression of cancer (Park et al., 2013). SIRT7, on the other hand, acts as a histone desuccinylase that is functionally associated with chromatin compaction and the maintenance of genome stability (Li et al., 2016) (Figure 2). The process of desuccinylation catalyzed by SIRT7 predominantly occurs within the nucleus and plays a crucial role in the DNA damage response and the survival of cells (Li et al., 2016).

As previously stated, the maintenance of a delicate equilibrium between succinylation and desuccinylation processes is of utmost importance for the regulation of physiological functions (Wang and Lin, 2021). Disruption of this equilibrium can lead to the development of various diseases, including inflammatory disorders, IR injury, and cancer (Choudhary et al., 2014; Sabari et al., 2017; Yang et al., 2017). The intricate involvement of SIRT5 in the process of carcinogenesis is currently under investigation by researchers (Yang et al., 2017) (Figure 2). It has been proposed that desuccinylation events facilitated by SIRT5 may play a role in the initiation and progression of tumorigenesis. The inhibition of SIRT5 has demonstrated a reduction in cell proliferation and tumor growth, implying that hyper-succinylation may exert similar effects on tumor growth by modulating SIRT5 (Li et al., 2015; Xiangyun et al., 2017). Furthermore, mounting evidence suggests that lysine succinylation is abnormally elevated during cancer development, indicating that succinylation regulates tumor energy metabolism (Song et al., 2017). Mutations in isocitrate dehydrogenase isoform 1 that impede SDH result in an elevated level of succinyl-CoA, thereby promoting cancerous metabolism (Li et al., 2015). The augmentation of succinylation under hypoxic conditions may potentially counteract the effects of IR injury in both cardiac and cerebral tissues. The proficient regulation of succinylation holds promise for unveiling novel therapeutic strategies for managing IR injury and potentially facilitating transplantation procedures (Murphy and O'Neill, 2018). In summary, comprehending the tissue-specific mechanisms implicated is imperative for the advancement of pharmacological interventions targeting succinylation and de-succinylation in hypoxia-related ailments such as inflammation, IR injury, and cancer.

## 4 Succinate and SDH in IR injury

IR injury is commonly observed when the blood supply to a solid organ, such as the heart, brain, lung, or kidney, is disrupted, subsequently followed by reperfusion in specific pathological conditions like heart attack, ischemic stroke, kidney IR injury, or organ transplantation (Kula-Alwar et al., 2019). The underlying mechanism of this injury primarily involves the generation of ROS by mitochondria, which triggers a cascade of events including aberrant immune responses, accumulation of succinate, and cellular harm (Yellon and Hausenloy, 2007; Murphy and Steenbergen, 2008; Burwell et al., 2009; Eltzschig and Eckle, 2011; Timmers et al., 2012) (Figure 1). During the state of ischemia, the absence of oxygen induces a reduction in mitochondrial respiration and a rise in the accumulation of succinate. Subsequent reperfusion prompts the swift oxidation of the accumulated succinate by SDH. Reverse electron transport (RET) through mitochondrial complex I allows some of the electrons in the mitochondria to break away from, ETC, leading to an inadequate reduction reaction of oxygen, which in turn drives the production of ROS that inflict oxidative harm upon cellular constituents such as lipids, proteins, and DNA (Figure 1). This oxidative damage serves to intensify the inflammatory response, thereby precipitating tissue injury and dysfunction. The significance of succinate and SDH’s involvement in the pathogenesis of IR injury underscores the potential of targeting succinate metabolism as a therapeutic strategy for the prevention and treatment of this condition (Panconesi et al., 2022). The development of pharmaceuticals that specifically target succinate metabolism may offer a novel approach to address the clinical challenge posed by IR injury.

SDH is a key enzyme involved in succinate formation during ischemia and its oxidation upon reperfusion (Davidson et al., 2019). Malonate, a competitive inhibitor of SDH, has emerged as a candidate therapy for selective SDH inhibition to reduce reperfusion injury. The protective effect of malonate was demonstrated using the malonate prodrug dimethyl malonate, which was effective when administered before and throughout ischemia (Chouchani et al., 2014). Adequate studies have shown that IR injury can be regulated through the accumulation of succinate during ischemia, which is subsequently re-oxidized by SDH to generate mitochondrial ROS (Chouchani et al., 2014). Succinate, therefore, acts as a metabolic signal of IR injury, responsible for generating mitochondrial ROS (Figure 1). Blocking ischemic succinate accumulation resulting from oxidation can abolish IR injury (Pell et al., 2016). Similar studies have demonstrated that preventing succinate from oxidation may decrease IR injury and increase tolerance in the process of liver transplantation (LT) from steatosis liver donors (Evans et al., 2008). By targeting succinate metabolism, malonate may provide additional therapeutic strategies for heart damage underlying chronic heart failure (Pell et al., 2016). In addition to malonate, it has been determined that ginsenoside Rb1 possesses the ability to mitigate myocardial IR injury by inhibiting the production of ROS originating from mitochondrial complex I according to a proteomic analysis (Jiang et al., 2021). The cerebral IR injury results in the ischemic accumulation of succinate, which subsequently induces Cdc42 succinylation and inhibits the proliferation of neural stem cells (Huang et al., 2023).

However, despite promising results *in vitro* and animal models, translation to human trials has proved challenging, with high failure rates due to low drug exposure at the target site or clinical safety issues. Translation failure in IR injury is likely caused by delivery difficulties or insufficient knowledge of the pathological mechanisms, leading to inappropriate drug targets. Therefore, further research is needed to develop effective and safe therapeutic strategies for targeting succinate metabolism in IR injury (Dambrova et al., 2021).

## 5 Succinate and SDH in tumor

Metabolites originating from the TCA cycle and respiratory enzymes, specifically succinate and SDH, have been found to have a significant impact on the initiation and progression of tumorigenesis (Mullen and DeBerardinis, 2012; Dalla Pozza et al., 2020; Kes et al., 2020). Notably, alterations in SDH activity leading to the accumulation of succinate, such as SDH mutations, have been observed (Dalla Pozza et al., 2020). Furthermore, extensive investigation has been conducted to elucidate the role of succinate in the development of cancer, encompassing its capacity to induce epigenetic and metabolic modifications, as well as its influence on cell migration, invasion, and angiogenesis.

### 5.1 Succinate and SDH in cancer-immune cycle

Succinate has garnered growing recognition for its multifaceted involvement in both immune and cancer-related processes, in addition to its established role in immuno-inflammatory responses. The cumulative evidence from numerous interconnected studies has consistently demonstrated that succinate possesses the capacity to regulate tumorigenesis within specific intrinsic microenvironments. Consequently, succinate is now acknowledged as a classical tumorigenic signal, alongside its well-established association with inflammation (Huang and Millar, 2013; Jiang and Yan, 2017).

SDH mutations and associated succinate pathways have been demonstrated to contribute to tumorigenesis (Bardella et al., 2011). Specifically, mutations in the SDH gene have been observed to expedite tumor development and can be detected in tumor tissues and cells (Pasini and Stratakis, 2009; Gaude and Frezza, 2014). The SDH enzyme plays a crucial role in the TCA cycle by facilitating the conversion of succinate to fumarate. Mutations in the SDH gene have been identified in various tumors, establishing SDH as a recognized tumor suppressor (Bardella et al., 2011; Wallace, 2012; Wu and Zhao, 2013; Jiang and Yan, 2017). However, mutations in this gene can result in succinate accumulation, which in turn stabilizes HIF-1α and fosters tumor growth (Selak et al., 2005) (Figure 1). Hence, it is imperative to engage in a comprehensive examination of succinate accumulation in the context of cancer. Succinate accumulation can manifest through diverse pathways that contribute to the development of tumors and immune responses. The regulation of SDH activity plays a crucial role in the buildup of succinate. Notably, the presence of a mutation in the gene responsible for encoding SDH in certain cancer types has been observed to diminish SDH activity, resulting in succinate accumulation and subsequent augmentation of mitochondrial ROS generation (Drose, 2013) (Figure 1). Hence, the functional deficiency of subunits in SDH resulting from mutation and carcinogenesis has garnered significant attention in numerous studies on metabolism (Tomitsuka et al., 2010). Furthermore, the function of SDH is reliant on the presence of oxidized FAD and NAD^+^ as cofactors (Gill, 2012). However, under conditions of hypoxia where these cofactors are predominantly in their reduced forms, SDH function is hindered, leading to the accumulation of succinate in the cancer-immunity cycle (Jiang and Yan, 2017). It is noteworthy to mention that the accumulation of succinate in M1 macrophages is closely associated with the activation of toll-like receptor 4 (TLR4) signaling. Upon activation by LPS, TLR4 signaling is triggered, resulting in the disruption of the Krebs cycle (O'Neill and Pearce, 2016) (Figure 1). This disruption subsequently leads to metabolic reprogramming and the accumulation of succinate. Previous research has demonstrated that LPS-activated macrophages induce succinate accumulation by inhibiting the activity of the PHD enzyme, thereby stabilizing HIF-1α (Figure 1). The stabilization of target genes responsible for encoding glycolytic enzymes and the pro-inflammatory cytokine IL-1β, which can impede tumor cell growth, enhance tumor survival, and intensify inflammation, is facilitated by this process (O'Neill and Pearce, 2016; Selak et al., 2005; Liu et al., 2016; Aspuria et al., 2014) (Figure 1). Succinate, in aggregate, may possess two distinct functions in the development of tumors, either promoting or hindering tumor growth. Nevertheless, thus far, SDH has exclusively demonstrated its involvement in suppressing tumor growth and advancement.

In the context of the cancer-immune cycle, succinate has the potential to exert an influence on the immune responses of various immune cells, including APCs and T cells. Notably, succinate has been observed to enhance the antigen presentation capacity of DCs by eliciting an adaptive response and impeding tumor growth (Palucka and Banchereau, 2012). Furthermore, upon encountering a sequence of stimuli originating from antigens, heightened activation of antigen-specific T cells can result in the secretion of cytokines such as TNF-α and interferon-γ (IFN-γ) during immune activation (Cortese et al., 2014) (Figure 1). These cytokines possess the ability to impede the progression of cancer cells and extend the survival of patients (Hadrup et al., 2013; Jiang and Yan, 2016).

### 5.2 Succinate and SDH in tumorigenesis

Po-Lin Tseng et al. have provided evidence indicating an inverse correlation between the malignancy of hepatocellular carcinoma (HCC) and the expression level of SDHB. Specifically, a higher degree of malignancy in HCC is associated with a lower expression of SDHB, which is significantly associated with advanced tumor stage and unfavorable prognosis (Tseng et al., 2018). Furthermore, in mouse models, experimental interventions involving the silencing and overexpression of SDHB have demonstrated the potential feasibility of regulating HCC growth and metastasis. Thus, the downregulation of SDHB expression in human HCC leads to a metabolic shift from aerobic respiration to glycolysis and the induction of the Warburg effect, ultimately facilitating tumor malignancy (Tseng et al., 2018). Studies have demonstrated that the reduction in SDH activity, resulting from the silencing of one of its subunits, promotes HCC cell proliferation and metastasis both *in vitro* and *in vivo* by increasing ROS and subsequently activating NF-κB signaling (Li et al., 2019). These findings suggest that SDH could potentially be targeted for therapeutic intervention in the treatment of HCC due to its tumor-suppressive role.

In addition to its association with HCC, the most closely correlated association with SDH is hereditary paraganglioma/phaeochromocytoma syndrome (HPGL/PCC), which is characterized by the presence of germline loss-of-function mutations in SDH genes (Brière et al., 2005). Various mechanisms of carcinogenesis have been suggested, as patients with HPGL/PCC have been found to harbor germline mutations in SDHB, SDHC, and SDHD (Eniafe and Jiang, 2021; Takács-Vellai et al., 2021). In addition to HPGL/PCC, a multitude of neuroendocrine and non-neuroendocrine neoplasms, such as gastrointestinal stromal tumors (GISTs), renal tumors, and thyroid tumors, have been discovered to exhibit a significant correlation with SDH gene mutations, alongside HPGL/PCC. The growing body of research highlights the tumor suppressor function of SDH and the oncogenic role of succinate as a metabolite in the progression of cancer (Neppala et al., 2019; Ibrahim and Chopra, 2020; Yong et al., 2020). The current research focuses on investigating the genetic and molecular mechanisms that lead to succinate accumulation as a result of SDH mutations, and how these mutations initiate neoplasm invasion and metastasis. Additionally, variants of SDH genes have been identified in thyroid C-cell hyperplasia and papillary thyroid cancers, independent of SDH mutations. Nevertheless, the importance of succinate and SDH in the development of cancer has been firmly established, resulting in the classification of succinate as an oncometabolite and SDH as a tumor suppressor.

## 6 Succinate and SDH as cancer biomarkers and treatment strategies

As previously stated, succinate serves as an oncometabolite, suggesting that the identification of these oncometabolites in cancer specimens from patients holds the potential to facilitate cancer detection, tumor screening, and comprehensive follow-up during the early phases of cancer (Semeraro et al., 2018). Furthermore, mass spectrometry and nuclear magnetic resonance technologies present promising methodologies that clinical practitioners can use to identify the buildup of succinate. Moreover, the utilization of immunohistochemistry (IHC) presents an opportunity to assess oncometabolites as potential cancer biomarkers in individuals afflicted with cancer (Yong et al., 2020). The abnormal expression of SDH, specifically the loss of the B subunit (SDHB), is implicated in the pathogenesis of neuroendocrine tumors as a prognostic factor, likely due to the accumulation of succinate in cancer (Hwang et al., 2014; Milione et al., 2017). A 2020 review by Dalla Pozza et al. provided a comprehensive summary of the detection of succinate and SDH as cancer biomarkers across various tissues (Dalla Pozza et al., 2020).

The deregulation of Complex II has the potential to result in an overabundance of ROS, which can induce apoptotic cell death in a manner specific to tumors. Consequently, numerous compounds that impact SDH activity have been examined for their potential as anticancer agents (Kluckova et al., 2013; Hwang et al., 2014) (Table 1). These compounds exhibit the ability to selectively target cancer cells by promoting apoptosis mediated by ROS, while preserving normal cells. Nevertheless, further investigation is necessary to comprehensively comprehend the underlying mechanisms and ascertain the effectiveness and safety of these compounds as potential anticancer treatments.

**TABLE 1:** Several compounds affecting SDH activity have been tested for their anticancer properties, IR injury and anti-inflammatory effects.

Compound	Target	Mechanism of action	Functions in cancer	Diseases	Ref
α-TOS	SDHC&SDHD	Interacting with both proximal (Qp) and distal (Qd) UbQ sites	Inducing cancer cells apoptosis via ROS	Colon cancer, breast cancer, prostate cancer, lung cancer, mesotheliomas, melanomas	Neuzil et al. (2001); Malafa et al. (2002); Weber et al. (2002); Kline et al. (2004); Tomasetti et al. (2004); Quin et al. (2005); Stapelberg et al. (2005); Malafa et al. (2006); Neuzil et al. (2006); Wang et al. (2007); Dong et al. (2008); Dong et al. (2009)
MitoVES	SDH	Qp site	Inducing cancer cells apoptosis via ROS	Breast carcinoma, colon cancer	Dong et al. (2011a); Dong et al. (2011b)
3-BP	HK		Causing cancer cells death by the fast depletion of ATP	Advanced cancers Fibrolamellar	Ko et al. (2004); Mathupala et al. (2006); Pereira da Silva et al. (2009); Ko et al. (2012); Pedersen (2012); Rodrigues-Ferreira et al. (2012)
	SDH		Inhibiting SDH activity	HCC	
3NP	SDH		Inducing ROS and apoptosis	Neuroblastoma, PD, HD	Huang et al. (2006); Gomez-Lazaro et al. (2007)
TTFA	SDHC&SDHD	Interfering with the UbQ-binding site	Inducing apoptosis and trigger superoxide/hydrogen peroxide and increase Ca^2+^ levels	Leishmania spp infection	Mehta and Shaha (2004)
Troglitazone	SDH		Interfering with SDH activity; Inducing cancer cell cycle arrest, differentiation, and apoptosis	Liposarcomas, prostate cancer, colorectal cancer	Demetri et al. (1999); Hisatake et al. (2000); Gupta et al. (2001); Kopelovich et al. (2002); Koeffler (2003); Soller et al. (2007); Wei et al. (2009)
Atpenins	SDH	UbQ	Interfering with the UbQ site, prevents reduction of UbQ and induces ROS	IR injury	Miyadera et al. (2003); Horsefield et al. (2006); Wojtovich and Brookes (2009); Ralph et al. (2011); Quinlan et al. (2012)
LND	SDHC&SDHD	Transferring electrons from the iron sulfur clusters to ubiquinone	Inducing ROS and cancer cell death	Melanoma	Guo et al. (2016)
DT-010	Complex II		Inducing ROS generation, cytotoxicity and promoting cell arrest	Breast cancer	Wang et al. (2016)
Chrysin	Complex II		Inhibiting SDH activity and increasing ROS generation associated to apoptosis	CLL	Salimi et al. (2017)
Dimethyl malonate	SDH		Inducing ROS with calcium dysregulation and ATP depletion via inhibiting SDH	IR injury (heart attack, ischemic stroke), tissue damage, and inflammation	Chouchani et al. (2014); Xu et al. (2018); Kula-Alwar et al. (2019)
Ginsenoside Rb1	Complex I		Inhibiting ROS production	Myocardial IR injury	Jiang et al. (2021)
3-BrPA	SDH		Decreasing SDH expression; Cancer cell cycle arrest and apoptotic induction via suppressing aerobic glycolysis	HCC, PC, endometrial cancer, *Staphylococcus aureus* infection; metastatic prostate cancer	Liu et al. (2009); Pereira da Silva et al. (2009); Cardaci et al. (2012); Birsoy et al. (2013); Byrne et al. (2014); Chapiro et al. (2014); Yadav et al. (2017); Abdel-Wahab et al. (2019); Pichla et al. (2019); Visca et al. (2019); Sun et al. (2020); Yu et al. (2021)
Compound 968, CB-839 and JQ1	Succinate	Inhibiting glutaminase 1 and BET	Suppressing the growth of the SDHB knockout cells	Colon cancer	Kitazawa et al. (2017); Godel et al. (2021)

α-TOS, α-tocopheryl succinate; SDH, succinate dehydrogenase; ROS, reactive oxygen species; MitoVES, mitochondrially targeted vitamin E succinate; 3-BP, 3-Bromopyruvate; HK, hexokinase; ATP, adenosine triphosphate; 3NP, 3-nitropropionic acid; TTFA, thenoyltrifluoroacetone; LND, lonidamine; DT-010, a conjugate of danshensu (DSS) and tetramethylpyrazine (TMP); 3-BrPA, 3-bromopyruvate; IR, injury, ischemia/reperfusion injury; PD, Parkinson’s disease; HD, Huntington’s disease; HCC, hepatocellular carcinoma; PC, pancreatic cancer; BET, bromodomain and extra-terminal; CLL, chronic lymphocytic leukemia; Compound 968 and CB-839, glutaminase 1 inhibitor; JQ1, a BET, inhibitor.

The identification of diverse pathways contributing to succinate accumulation presents promising avenues for the regulation of succinate levels and the modulation of tumorigenesis. The manipulation of succinate levels holds potential for the development of innovative cancer therapies. By comprehending the underlying molecular mechanisms governing succinate accumulation and its consequential impact on tumorigenesis, it becomes feasible to devise novel therapeutic approaches that specifically target succinate metabolism in malignant cells. These therapeutic interventions have the potential to enhance patient prognoses and offer efficacious treatment alternatives for diverse cancer types. Further investigation is warranted to comprehensively elucidate the precise involvement of succinate in cancer and to devise targeted therapeutic approaches that proficiently modulate its concentrations.

## 7 Conclusion and future perspectives

Historically, succinate and SDH have been recognized as pivotal contributors to ATP generation in the context of mitochondrial energy metabolism. Nevertheless, it has become evident that comprehending their extensive involvement in IR injury, immuno-inflammatory responses, and tumorigenesis could offer innovative and potent insights into disease control (Grimolizzi and Arranz, 2018). The diverse mechanisms involving succinate and SDH in the process of neoplasm invasion and metastasis imply that the identification of succinate as an oncometabolite could serve as a valuable diagnostic indicator for tracking tumors, whereas SDH, acting as a tumor suppressor, may present a promising target for anticancer treatment. Nevertheless, despite the potential of succinate accumulation and abnormal succinylation expression as diagnostic markers, the majority of existing studies have been conducted in laboratory settings, and it is crucial to validate ideal and efficacious molecular markers *in vivo*. On the other hand, despite the evident effectiveness of numerous compounds *in vitro* and animal models in targeting succinate to prevent ischemia-reperfusion injury and tumorigenesis, the translation of these findings from laboratory experiments to clinical application is fraught with challenges. Primarily, the attainment of the necessary concentrations of these compounds for protective and preventive purposes may present difficulties. Additionally, the development of strategies to enhance the delivery of succinate metabolism-targeting therapies to cells will be crucial in overcoming these obstacles (Gupta et al., 2021). The precise timing of targeted therapy administration holds significant importance in mitigating adverse reactions and ensuring optimal drug concentration within the affected tissue. Additionally, comprehending the pharmacokinetics of succinate-based medications during administration is crucial for establishing appropriate drug delivery schedules. Moreover, attaining selectivity towards specific tissues is imperative to prevent off-target effects. Overcoming these challenges is indispensable for the successful transition of succinate-based therapeutics from experimental settings to clinical application in the foreseeable future.

To summarize, there has been a comprehensive discourse on the role of succinate and SDH in the aforementioned areas of interest. Future investigations should persist in exploring potential diagnostic and therapeutic strategies that focus on succinate metabolism for inflammation, ischemia-reperfusion injury, and tumors. Additional research is imperative to validate the associations and underlying mechanisms between succinate and SDH in metabolic alterations as signaling pathways in the regulation of immune cells, potentially paving the way for innovative therapeutic interventions.
